# Transgelin increases metastatic potential of colorectal cancer cells in vivo and alters expression of genes involved in cell motility

**DOI:** 10.1186/s12885-016-2105-8

**Published:** 2016-02-04

**Authors:** Hui-min Zhou, Yuan-yuan Fang, Paul M. Weinberger, Ling-ling Ding, John K. Cowell, Farlyn Z. Hudson, Mingqiang Ren, Jeffrey R. Lee, Qi-kui Chen, Hong Su, William S. Dynan, Ying Lin

**Affiliations:** Guangdong Provincial Key Laboratory of Malignant Tumor Epigenetics and Gene Regulation, Sun Yat-sen Memorial Hospital, Sun Yat-sen University, Guangzhou, 510120 China; Department of Gastroenterology and Hepatology, Sun Yat-sen Memorial Hospital, Sun Yat-sen University, Guangzhou, 510120 China; Department of Gastroenterology and Hepatology, The First Affiliated Hospital, School of Clinical Medicine of Guangdong Pharmaceutical University, Guangzhou, 510000 China; Center for Biotechnology and Genomic Medicine, Georgia Regents University, Augusta, GA 30912 USA; GRU Cancer Center, Georgia Regents University, Augusta, GA USA; Department of Anatomy, Wu Han University, Wuhan, China; Institute of Molecular Medicine and Genetics, Georgia Regents University, Augusta, GA USA; Department of Pathology, Georgia Regents University, and Charlie Norwood Veterans Affairs Medical Center, Augusta, GA USA; Departments of Radiation Oncology and Biochemistry, Emory University, Atlanta, GA USA

**Keywords:** Transgelin, Colorectal cancer, Experimental metastasis, Gene regulation, Invasiveness, Biomarker

## Abstract

**Background:**

Transgelin is an actin-binding protein that promotes motility in normal cells. Although the role of transgelin in cancer is controversial, a number of studies have shown that elevated levels correlate with aggressive tumor behavior, advanced stage, and poor prognosis. Here we sought to determine the role of transgelin more directly by determining whether experimental manipulation of transgelin levels in colorectal cancer (CRC) cells led to changes in metastatic potential in vivo.

**Methods:**

Isogenic CRC cell lines that differ in transgelin expression were characterized using in vitro assays of growth and invasiveness and a mouse tail vein assay of experimental metastasis. Downstream effects of transgelin overexpression were investigated by gene expression profiling and quantitative PCR.

**Results:**

Stable overexpression of transgelin in RKO cells, which have low endogenous levels, led to increased invasiveness, growth at low density, and growth in soft agar. Overexpression also led to an increase in the number and size of lung metastases in the mouse tail vein injection model. Similarly, attenuation of transgelin expression in HCT116 cells, which have high endogenous levels, decreased metastases in the same model. Investigation of mRNA expression patterns showed that transgelin overexpression altered the levels of approximately 250 other transcripts, with over-representation of genes that affect function of actin or other cytoskeletal proteins. Changes included increases in HOOK1, SDCCAG8, ENAH/Mena, and TNS1 and decreases in EMB, BCL11B, and PTPRD.

**Conclusions:**

Increases or decreases in transgelin levels have reciprocal effects on tumor cell behavior, with higher expression promoting metastasis. Chronic overexpression influences steady-state levels of mRNAs for metastasis-related genes.

**Electronic supplementary material:**

The online version of this article (doi:10.1186/s12885-016-2105-8) contains supplementary material, which is available to authorized users.

## Background

Colorectal cancer (CRC) is a leading cause of cancer death worldwide. Although early-stage, localized CRC is often curable by surgical resection, some of these patients will experience recurrent, metastatic disease. Currently, the best predictor of risk is lymph node status. There is, however, considerable interest in identifying mechanistically based, molecular markers to improve the ability to forecast individual risk of disease recurrence. In a prior study, we sought to identify such markers by proteomic analysis of samples obtained by laser capture micro-dissection of tumor tissue from node-negative and node-positive patients. Of these, transgelin, a 23 kDa actin binding protein, ranked the highest in a statistical analysis [1].

Transgelin, also known as SM22α, is an abundant protein in normal tissue [2]. It promotes podosome formation [3] and contributes to cell motility in response to injury or inflammation [4, 5]. Transgelin knockout mice are viable and fertile [6], but exhibit reduced smooth muscle contractility [7, 8] and enhanced atherogenesis in a susceptible background [9].

Perhaps because of its abundance, transgelin has been frequently identified in proteomic profiling studies of cancer. Early studies showed that expression is down-regulated in early-stage cancer models [10, 11], leading to the idea that transgelin is a tumor suppressor (reviewed in [12]). However, proteomic studies of transgelin in human cancers often indicate up-regulation in aggressive, late-stage disease [13–16]. Several studies, although not all, have shown a correlation between higher tumor transgelin levels and aggressive behavior, advanced stage, or poor prognosis [1, 17–21]. Consistent with this, various studies have suggested an influence of transgelin on motility or invasiveness in cell-based models [1, 22, 23]. A recent review summarizes current understanding of transgelin in normal tissue and malignancy [24].

One hypothesis that fits with most of the experimental and clinical data is that transgelin is not a marker of cancer *per se*, but rather a marker of metastatic potential in advanced disease. One way that transgelin may influence metastasis is through direct interaction with cytoplasmic actin. There is also evidence that manipulation of transgelin expression levels affects the expression of other metastasis-related genes [1], suggesting a possible dual mechanism of action.

Here, we adopt an approach based on comparison of isogenic CRC cell populations that differ in transgelin expression but are otherwise identical. We generated new isogenic cell pairs, in which low transgelin-expressing CRC cell lines were transfected with a transgelin cDNA vector to create high-expressing derivatives. We also further characterized a previously created isogenic cell pair, in which high transgelin-expressing cells were stably transfected with miRNA to attenuate transgelin expression. Overexpression in low-expressing cells and attenuation in high-expressing cells had reciprocal effects on cell behavior. In addition, comprehensive gene expression profiling showed that increasing transgelin expression in a low-expressing background led to changes in expression of ~250 other mRNAs. Thus, experimental manipulation of transgelin levels leads to wide-scale transcriptional reprogramming.

## Results

### Establishment of a new transgelin overexpression cell model

Previously, we described stable transfection of HCT116 and SW480 CRC cells with transgelin miRNA to create two independent cell line pairs that differ in transgelin protein and mRNA levels [1]. In both cases, lower transgelin expression was associated with reduced motility, invasiveness, and resistance to anoikis. This phenotype could furthermore be reversed by transfection with miRNA-resistant transgelin cDNA [1].

**Fig. 1:**
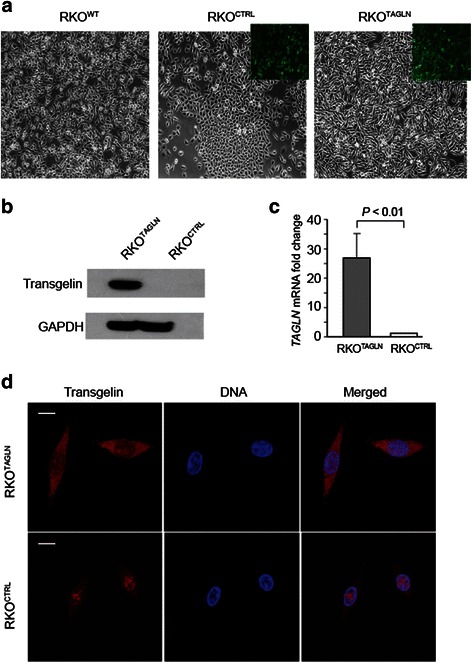
Establishment of transgelin-overexpressing RKO cell lines. **a**. Phase contrast images of unmodified RKO cells (RKO^WT^) and derivatives stably transfected with empty vector (RKO^CTRL^) or with transgelin cDNA (RKO^TAGLN^). Inset, confocal fluorescence imaging showing the transfection marker, EmGFP. **b**. Immunoblot analysis of total cell extracts from RKO^CTRL^ or RKO^TAGLN^ cells. **c**. Fold change in transgelin mRNA expression as determined by real-time PCR analysis, normalized to GAPDH. Mean of three experiments, error bars denote standard deviation. *P* value determined by Student’s t-test. **d**. Anti-transgelin immunostaining of RKO^CTRL^ or RKO^TAGLN^ cells. Cells were counterstained for DNA with DAPI; merged image is indicated. Scale bar, 5 μm

To extend and confirm the results of the transgelin knockdown experiments, we tested a complementary approach, creating a new isogenic cell line model in which transgelin was overexpressed in CRC cells that express endogenous transgelin at very low levels. We transfected RKO CRC cells, in which endogenous transgelin is nearly undetectable, with a transgelin cDNA and selected stable transfectants. After selection, virtually all cells expressed a fluorescent transfection marker (Fig. 1a). Immunoblotting showed high levels of transgelin protein in cells that received the transgelin cDNA (RKO^*TAGLN*^), whereas transgelin remained undetectable in cells that received an empty control vector (RKO^*CTRL*^) (Fig. 1b). Measurement of the relative levels of transgelin mRNA by qPCR showed an increase of about 25-fold (Fig. 1c). The overexpressed transgelin was distributed primarily in the cytoplasm, as indicated by immunofluorescence staining (Fig. 1d).

### Effects of transgelin on invasiveness, clonogenicity, and anchorage-independent growth

**Fig. 2:**
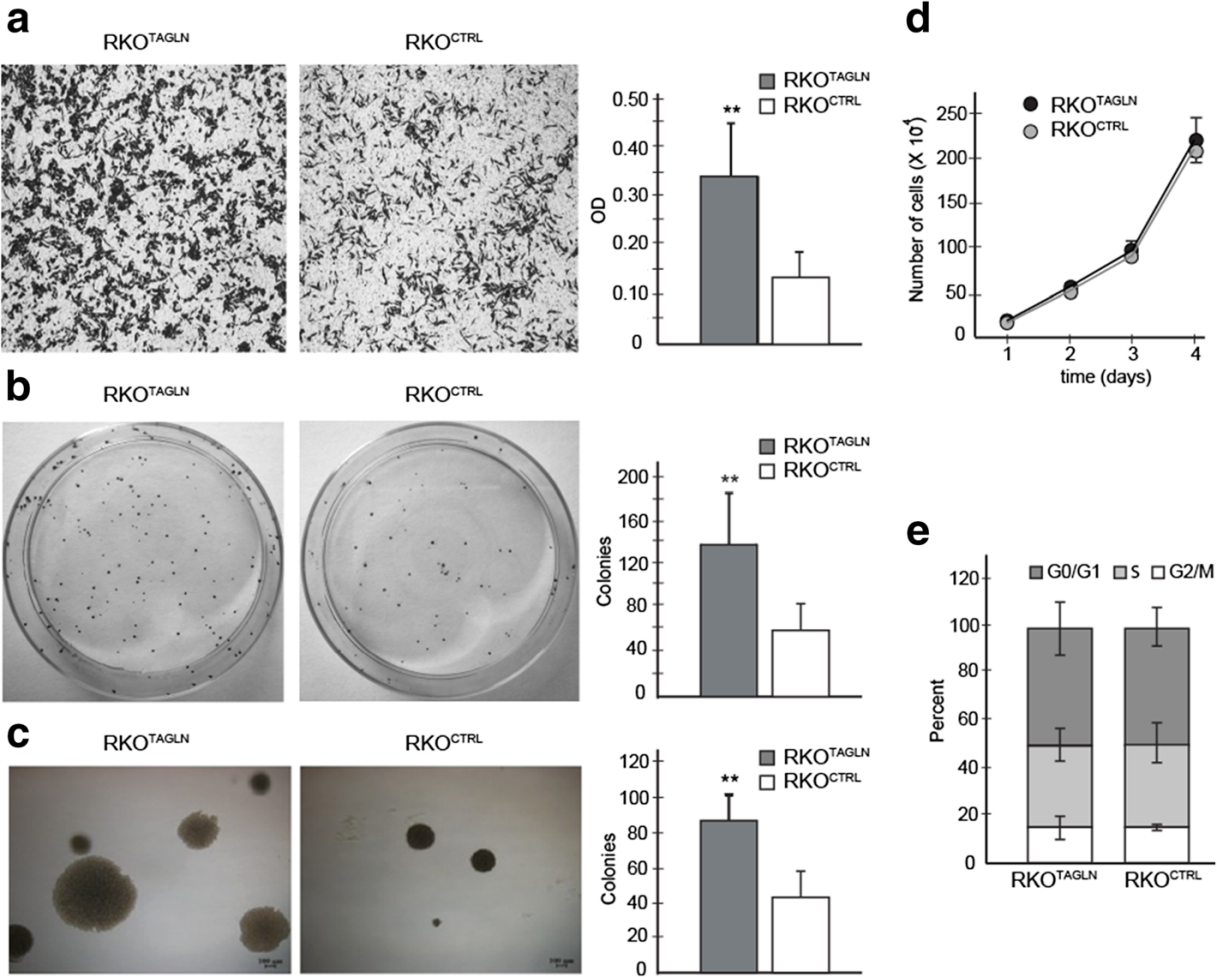
Effects of *TAGLN* overexpression on in vitro cell behavior. **a**. Invasiveness. Left, representative images showing invasion of RKO^TAGLN^ and RKO^CTRL^ cells through Matrigel-coated Transwell filters, right, quantification of filter staining. **b**. Clonogenicity. Left, representative images of plates seeded with RKO^TAGLN^ or RKO^CTRL^ cells, right, quantification of colony formation after 12 days. **c**. Growth in soft agar. Left, representative images of colonies formed by RKO^TAGLN^ and RKO^CTRL^ cells, right, quantification colony formation after 17 days. **d**. Cell proliferation. Graph shows cell count in replicate cultures of RKO^TAGLN^ and RKO^CTRL^, counted daily for four days. Graphs in panels **a**-**d** show mean of three experiments. Error bars denote standard deviation. **e**. Cell cycle distribution. Graph shows the percentage of RKO^TAGLN^ and RKO^CTRL^ cells in G0/G1, S, and G2/M phases of the cell cycle. Data are mean of technical replicates from a single representative experiment. Error bars denote standard deviation. ** *P* < 0.01 by Student’s t-test

We investigated the phenotype of the newly created RKO cell pair using in vitro assays. Transgelin overexpression led to a 2 to 3-fold increase in invasiveness in a Transwell assay (Fig. 2a). There was also an increase in the ability to form colonies when plated at low density (Fig. 2b), and in the number and size of colonies in a soft-agar growth assay (Fig. 2c). Differences were highly significant in all three assays (*P* < 0.01). Interestingly, transgelin expression had essentially no effect on growth rate or cell cycle distribution under standard cell culture conditions (Fig. 2d, e), suggesting that transgelin expression primarily affects behaviors relevant to metastasis (such as invasiveness, clonogenicity, and anchorage-independent growth) rather than growth *per se*.

### Effect of transgelin on experimental metastasis

We next tested the behavior of isogenic cell pairs in a model of experimental metastasis. RKO^*TAGLN*^ or RKO^*CTRL*^ cells were injected via the tail vein into *scid* mice. We also tested the behavior of previously described HCT 116 cells stably transfected with a transgelin miRNA knockdown vector (HCT116 ^*TAGLN-KD*^) or with empty control vector (HCT116^*CTRL*^) [1] in the same assay.

**Fig. 3:**
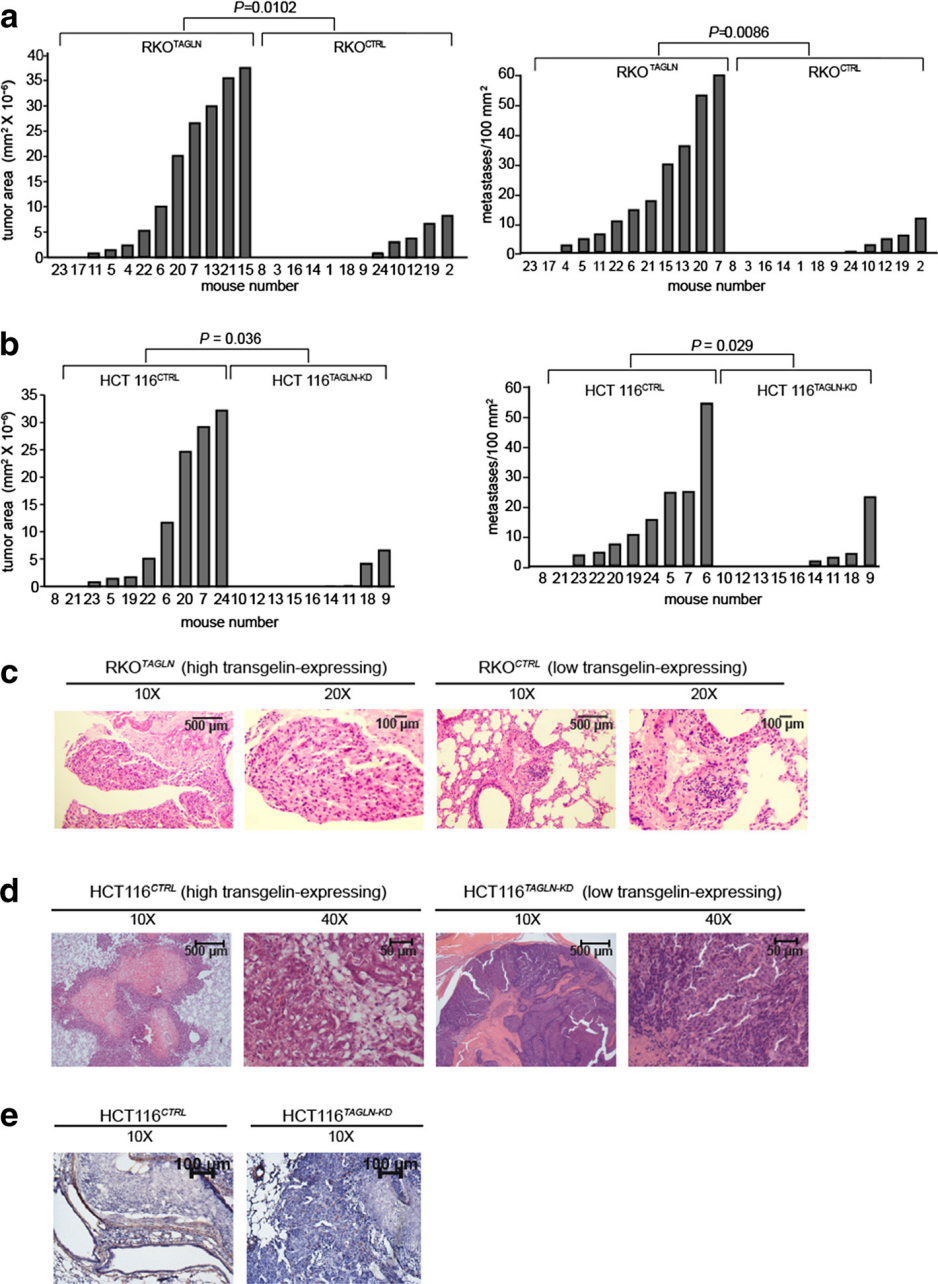
Experimental metastasis assay. Mice were injected with test cells via the tail vein as described in Materials and Methods. **a**. Aperio Precision image analysis on representative lung sections from animals injected with RKO cell derivatives. Twelve mice were used in each group. Left, total tumor area per lung section; right, number of metastases per unit area of lung tissue. *P* value reflects results of a non-parametric Wilcoxon rank sum test. **b**. Same analysis for HCT 116 cell derivatives. Ten mice were used in the HCT116^CTRL^ group and 9 were used in the HCT116^TAGLN-KD^ group. Statistical analysis as in Panel a. **c**. Histology of representative tumor sections from mice injected with RKO cell derivatives. **d**. Same for mice injected with HCT116^CTRL^ derivatives. HCT116^CTRL^-derived tumor is a lung metastasis, HCT116^TAGLN-KD^-derived tumor arose near the injection site. **e**. HCT116-derived tumors stained with anti-transgelin

Tumor burden in each experimental group was measured by quantitative histologic analysis. Mice receiving RKO^*TAGLN*^ cells had more tumors than those receiving RKO^*CTRL*^ cells, and the tumors occupied a greater fraction of the lung area (Fig. 3A). Similar results were seen with HCT116^*CTRL*^ and HCT116^*TAGLN-KD*^ cells (Fig 3B). In both instances, the member of the isogenic pair that had higher transgelin levels also had a greater tumor burden.

Although transgelin levels affected the number and size of metastases, there were no consistent differences in tumor histology (Fig. 3c, d). Immunostaining of HCT116-derived tumors with anti-transgelin antibody showed that tumors derived from the two cell populations retained their respective transgelin phenotypes in vivo, with no evidence of reversion (Fig. 3e). We did note that injection with HCT116 ^*TAGLN-KD*^ cells resulted in an unexpected incidence tumors near the injection site, instead of or in addition to the lung metastases (6/10 with HCT116 ^*TAGLN-KD*^ versus 1/10 with HCT116^*CTRL*^). Tumors near the injection site were not grossly evident in mice injected with the RKO cells.

### Gene expression profiling to identify downstream genes

Prior studies suggest that transgelin is capable of both activating and repressing genes involved in tumor progression [1, 25, 26], although only a small number of individual genes have been examined to date. To define the full scope of transgelin-mediated gene regulation, we performed comprehensive gene expression profiling on RKO^*CTRL*^ and RKO^*TAGLN*^ cells using Affymetrix microarray technology.

**Fig. 4:**
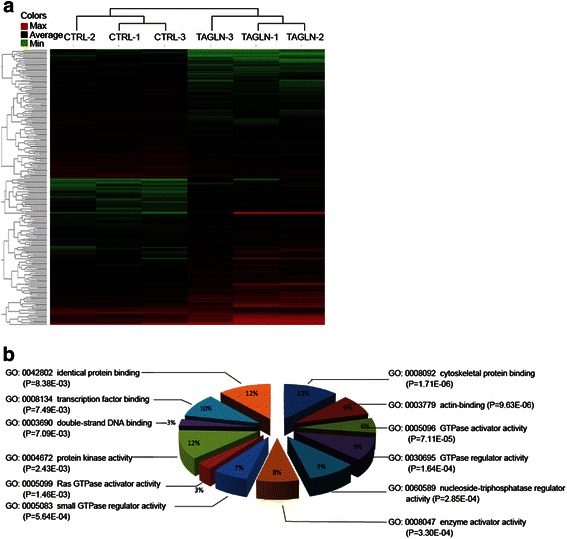
Effects of *TAGLN* overexpression on other genes. Gene expression profiling was performed using the Affymetrix platform. **a**. Hierarchical clustering analysis of 256 genes that show significant expression differences (*P* < 0.05) and a minimum 2-fold change between RKO^TAGLN^ and RKO^CTRL^ cells. Color represents relative gene expression on a log-transformed scale. **b**. Gene Ontology analysis for genes in Panel **a**

Based on criteria of adjusted *P* value <0.05 and a minimum 2-fold change, 256 transcripts were significantly affected, with approximately equal numbers of transcripts increased and decreased (Fig. 4a). The most significantly affected categories of genes were those involved in cytoskeletal and actin binding (Fig. 4b). Other categories that were significantly affected included GTPase regulatory activities, other enzyme regulatory activities and identical protein binding (the ability to form homodimers or higher-order multimers). Table 1 shows the ten most highly up-regulated and ten most highly down-regulated genes. Interestingly, prior studies implicate a number of these in metastasis or cell motility (see Discussion).

**Table 1:** Transcripts up- or down-regulated in transgelin cDNA-transfected RKO cells

Rank	Symbol	Gene name	Fold up	*P* value	Function
1	HOOK1	Hook homolog 1	95.7	0.011	actin binding
2	SDCCAG8	serologically defined colon cancer antigen 8	85.8	0.023	microtubule organizing center
3	ENAH	enabled homolog	17.7	0.045	actin binding
4	TNS1	Tensin 1	14.3	0.014	actin binding
5	ST8SIA4	ST8 alpha-N-acetyl-neuraminide alpha-2, 8-sialyltransferase 4	13.2	0.047	sialyltransferase activity
6	ARHGAP29	Rho GTPase activating protein 29	13.0	0.046	GTPase activator activity
7	FBXL14	F-box and leucine-rich repeat protein 14	11.3	0.005	protein binding
8	ZNF704	zinc finger protein 704	10.5	0.020	zinc ion binding
9	CCDC141	coiled-coil domain containing 141	8.7	0.036	protein binding
10	SPECC1	sperm antigen with calponin homology and coiled-coil domains 1	8.2	0.020	cell adhesion
Rank	Symbol	Gene name	Fold down	*P* value	Function
1	EMB	embigin	15.9	0.005	cell adhesion
2	WIPF1	WAS/WASL interacting protein family, member 1	13.0	0.006	actin binding
3	BCL11B	B-cell CLL/lymphoma 11B (zinc finger protein)	12.0	0.0004	nucleic acid binding
4	IL15	interleukin 15	11.3	0.010	signal transducer activity
5	PTPRD	protein tyrosine phosphatase, receptor type, D	11.0	0.021	phosphoprotein phosphatase
6	FRRS1	ferric-chelate reductase 1	10.4	0.004	ferric-chelate reductase
7	INSM1	insulinoma-associated 1	10.1	0.045	nucleic acid binding
8	PTGIS	prostaglandin I2 (prostacyclin) synthase	9.8	0.006	monooxygenase
9	PDGFB	platelet-derived growth factor beta polypeptide (simian sarcoma viral (v-sis) oncogene homolog)	9.8	0.004	PDGF receptor binding
10	POU3F1	POU class 3 home box 1	8.7	0.005	DNA binding

Gene expression profiling was performed on RKO cell populations that were stably transfected with empty control vector or with transgelin cDNA expression vector. Experiment was performed using Affymetrix microarray technology as described in Methods. *P* values have been adjusted for multiple testing

### Confirmation of gene expression changes by qPCR

**Fig. 5:**
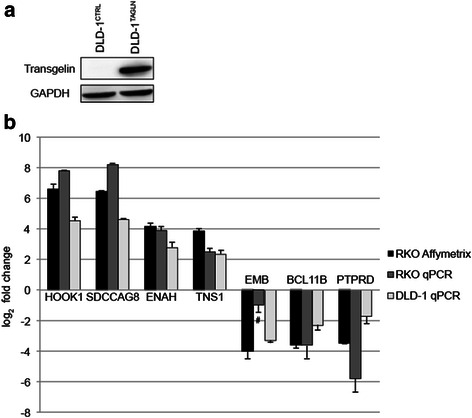
Expression of selected mRNAs determined by qPCR in RKO and DLD-1 cell derivatives. **a**. Immunoblot analysis of total cell extracts from DLD-1 derivatives stably transfected with empty vector (DLD-1^CTRL^) or with transgelin cDNA (DLD-1^TAGLN^). **b**. Real-time RT-PCR analysis for the levels of mRNA in RKO and DLD-1 cells of selected top-ranked differentially expressed genes identified by the cDNA microarray. Gene expression was normalized to GAPDH. Error bars denote standard deviation. All changes were significant (*P* < 0.05 by Student’s t-test) except for the one result indicated (#)

To confirm the microarray results and investigate their generality, we performed qPCR analysis to independently determine mRNA levels for seven genes, chosen from Table 1 based on cancer relevance (see Discussion for details on the function of individual genes). We performed the analysis using RNA from the RKO cells, which were used for microarray, and also from an isogenic cell pair derived from DLD-1 cells. Like RKO, DLD-1 is a widely studied human CRC line with low endogenous transgelin levels. Transgelin protein expression was undetectable by immunoblotting in cells stably transfected with empty control vector (DLD-1^*CTRL*^) and was greatly increased following transfection with the transgelin expression vector (DLD-1^*TAGLN*^) (Fig. 5a).

The effect of chronic transgelin expression in RKO and DLD-1 cells was strikingly similar and correlated well with the microarray data (Fig. 5b and Additional file 1: Table S1). HOOK1, SDCCAG8, ENAH, and TNS1 were up-regulated in the presence of transgelin, with effect sizes ranging from 5-fold to more than one hundred-fold. EMB, BCL11B, and PTPRD were down-regulated 3- to 60-fold. The significance of these changes in gene expression will be taken up in the Discussion; all of the genes are related to the cytoskeleton or cancer, and concordant regulation by transgelin in two independent CRC lines suggests a general effect with a common mechanism.

## Discussion

### Up-regulation of transgelin in low-expressing CRC cells and down-regulation in high-expressing cells have reciprocal effects

We characterized a new isogenic pair of high and low transgelin-expressing cell populations. In contrast to our previous study [1], where we knocked down expression in two high expressing CRC lines, here we increased transgelin expression in a low-expressing line. The overexpressing variant showed gain of metastasis-related behaviors in cell-based assays. Thus, overexpressing cells showed increased ability to invade Matrigel, an increased ability to form colonies when plated at low density, and more and larger colonies in soft agar. The reciprocal effects of up-regulation and down-regulation further support the idea that transgelin levels are causally related to metastatic behavior.

### Transgelin levels affect metastatic potential

Here, we show that experimental manipulation of transgelin expression levels affects the metastatic potential of CRC cells in vivo. In the experimental metastasis assay, control HCT116 cells and RKO cells with stable overexpression of transgelin produced more and larger tumors in the lung than their isogenic, low transgelin-expressing counterparts. The widely used tail vein assay provides a composite measure of the efficiency of sequential events required for metastasis, including survival of isolated cells in the bloodstream, extravasation into the lung, and subsequent colonization and proliferation to form observable tumors [27]. Each of these steps has a parallel counterpart that was previously measured in cell culture [1], including resistance to anoikis, invasiveness in a Transwell assay, and colony formation at low density, respectively. Although a number of proteomic studies (but not all) have shown a correlation between transgelin levels and advanced stage disease and poor prognosis, data here imply a direct causal relationship between transgelin levels and metastatic potential in vivo.

### Mechanism of the transgelin effect

In normal cells and tissue, transgelin interacts directly with the actin, influencing cell motility [3–5]. This mechanism may apply in cancer cells as well. In addition to direct interaction with actin, transgelin may also influence cell phenotype by affecting gene regulation. This idea is based on transgelin’s partial nuclear localization, its homology to transgelin 3, which has transcriptional regulatory activity [28], and other evidence [1, 25, 26]. To address the influence of transgelin on gene regulation in greater depth, we performed comprehensive gene expression profiling comparing low- and high-expressing RKO cells. Altering transgelin levels significantly affected the expression of a set of ~250 other genes, with statistical overrepresentation of cytoskeletal and actin-binding proteins, together with various other regulatory genes.

The five most-highly upregulated genes in Table 1 are connected with cancer, and in some cases specifically with tumor metastasis. HOOK1 (up-regulated 96-fold) interacts with microtubules and is up-regulated in breast cancer [29]. SDCCAG8 (up-regulated 86-fold) organizes the centrosome and is related to a colon cancer autoantigen [30]. ENAH (up-regulated 18-fold), which is the mammalian enabled homology and is also known as Mena, is a transcriptional target of the wnt/beta-catenin pathway [31]. It facilitates cell invasion via phosphatidylinositol 3-kinase-dependent local accumulation of actin filaments [32]. Increased ENAH/Mena expression levels correlate with invasiveness of breast and salivary gland tumors[33, 34], and are also seen in colorectal cancer and in polyps with high grade dysplasia [35]. TNS1 (up-regulated 14-fold), which is also known as Tensin 1, has actin cross-linking activity and localizes to focal adhesions. Prior studies show that increased expression of Tensin 1 correlates with tumor cell migration [36, 37]. A sialyltransferase (ST8SIA4) has been shown to promote metastatic dissemination in pancreatic cancer by interfering with E-cadherin dependent cell adhesion [38].

Of the most highly down-regulated genes, EMB (down-regulated 16-fold) is thought to mediate adhesion to the extracellular matrix [39]. BCL11B (down regulated 12-fold) is a tumor suppressor that is widely mutated in acute lymphocytic leukemia [40] and has recently been shown to be methylated, and thus likely down-regulated, in prostate cancers [41]. PTPRD (down-regulated 11-fold), a tumor suppressor that is frequently mutated in glioblastoma [42, 43], Ewing’s sarcoma [44], lung cancer [45], cutaneous squamous cell carcinoma [46], and laryngeal squamous cell carcinoma [47]. PTPRD has previously been shown to suppress colon cancer cell migration in cooperation with β-catenin/TCF signaling [48].

Based on an analysis of a more limited set of genes, we had previously hypothesized that transgelin regulates the epithelial-to-mesenchymal transition (EMT). In one cell line, fibronectin and vimentin (mesenchymal markers) and occludin (an epithelial marker) were affected. In another, only fibronectin was affected. In the RKO model, however, neither these nor other markers of EMT were altered. Thus the effect of transgelin on EMT, if any, appears to be cell-line specific.

It remains unclear whether the effect of transgelin on gene regulation occurs by a direct or indirect mechanism. Transgelin localized partially in the nucleus of the CRC cells; it shares 85 % similarity to *TAGLN3*, which is believed to be a transcriptional regulator [28]; Bioinformatic analysis using the DP-bind Web server (http://lcg.rit.albany.edu/dp-bind) identified several segments of transgelin as having high DNA-binding potential (data not shown); studies also suggests that nuclear actin-binding proteins participate widely in the transcription processes (reviewed in [49]). We were, however, unable, to detect direct association of transgelin with RNA polymerase II or chromatin (HMZ, YYF, and YL, unpublished results), which suggests that transgelin most likely works indirectly, through interaction with other transcriptional regulatory proteins, rather than by direct interaction with the polymerase or template.

### Transgelin in the context of human CRC

The original impetus for the studies reported here was to identify biomarkers that could be quantified at the time of surgical resection and used to predict metastatic risk. Transgelin is abundant in normal tissue and is not a marker of cancer *per se*. In our work, however, it discriminated well between node-positive and node-negative CRC specimens [1].

Evaluation of potential biomarkers should ideally include evidence that they influence the biological process of interest; that is, adding or subtracting transgelin in a given context influences phenotypic behaviors associated with metastatic risk. Combining the results presented with those of a previous study [1], we have created four isogenic pairs (HCT116, SW480, RKO, and DLD-1), three have been characterized with respect to in vitro phenotypes (HCT116, SW480, and RKO) and two (HCT116 and RKO) have been characterized in an animal model of experimental metastasis. The combined results are consistent with a contribution of transgelin to metastatic potential.

Human CRC is characterized by genomic instability and variability, and caution is warranted in generalizing from results with individual cell lines. However, laboratory findings provide a rationale for continuing to explore the potential of transgelin, perhaps in conjunction with other biomarkers, as a predictor of individual risk. As noted earlier, proteomic studies of human cancer are not in universal agreement with respect to the role of transgelin, and it will be important to identify the root cause of the discrepancies, which could relate either to different patient populations or to technical factors. Separately, the finding that a number of gene products are co-regulated with transgelin suggests that they may be attractive targets for drug therapy in advanced CRC.

## Conclusions

The results support a hypothesis that transgelin expression levels influence metastatic potential in CRC cell lines, and this may occur in part due to altered expression of downstream target genes that affect cell motility.

## Methods

### Isogenic cell line pairs

RKO and DLD-1 colon carcinoma cells were obtained from ATCC (Manassas, VA; CRL-2577 or CRL-221 respectively). Cells were transfected with pcDNA6.2/EmGFP-Bsd/V5-DEST or pcDNA6.2/EmGFP-Bsd/V5-TAGLN-mut vectors; the latter was generated using pDONR221-TAGLN-mut from a previous study[1] by an LR recombination). Stable transfectants were selected in medium containing 25 μg/ml blasticidin (Invitrogen, Carlsbad, CA). Immunoblot analysis was performed using anti-transgelin IgG or anti-GAPDH IgG1 (Abcam, Cambridge, MA) with ECL substrate for detection (Thermo Fisher Scientific, Rockford, IL). Immunofluorescence was performed on paraformaldehyde-fixed cells as described [1]. For qPCR, total RNA was extracted using Trizol (Invitrogen), cDNA was prepared, and qPCR was performed using primers in Additional file 2: Table S2. Reactions were performed using a SYBR Green PCR kit (Qiagen, Germantown, MD) and a LightCycler 480 instrument (Roche Applied Science, Indianapolis, IN).

Creation and characterization of HCT116^CTRL^ and HCT116^TAGLN-KD^ populations has been described [1]. In brief, cells were transfected with plasmid expressing an artificial microRNA (Invitrogen, Hmi416875) or with control vector and drug selection was applied to obtain cell populations that differed by about five-fold in transgelin expression levels.

### Transwell invasion assay

Cell invasion assays were performed as described [1, 50] using a Transwell permeable support (8.0 μm pore size in 6-well format, Corning Life Sciences, Corning, NY). Filters were coated with 650 μg/ml Matrigel (BD Biosciences, Sparks, MD) according to the manufacturer’s protocol. Cells were harvested and resuspended in serum-free medium, and 2 × 10^5^ cells were applied onto the upper chamber of the Transwell apparatus. The bottom chamber contained 0.6 ml of medium supplemented with 10 % fetal bovine serum. Cells were incubated for 48 hours. Cells that did not migrate were removed by cotton swabbing. Remaining cells were fixed with 4 % formaldehyde for 20 minutes at room temperature and stained with 0.1 % crystal violet for 15 minutes, also at room temperature. The stained cells were extracted with 10 % acetic acid, and the absorbance at 595 nm was measured.

### Clonogenic survival assay

Cells were plated at 2 × 10^2^ per 60 mm dish and incubated with complete growth medium for 12 days. Colonies were fixed and stained with staining buffer (0.1 % crystal violet, 4 % formaldehyde) for 30 minutes at room temperature.

### Soft agar colony formation assay

Eagle’s MEM supplemented with 20 % fetal bovine serum, was adjusted to 0.6 % agar and distributed in a 6-well plate to form a base layer. Cells (2.5 × 10^2^ cells per well) were suspended in the same medium containing 0.35 % agar and the suspension was dispensed to each well. Cultures were fed twice weekly with an overlay of complete medium. After 17 days colonies of 100 or more cells were counted.

### Cell proliferation assay

Replicate cultures were seeded in 6-well dishes (1 × 10^5^ cells/well). Triplicate samples of each cell population were harvested and counted daily for four days.

### Cell cycle analysis

For cell cycle analysis, 5 × 10^5^ cells were fixed with 70 % ethanol overnight at 4 °C, washed twice with PBS, and incubated with 50 μg RNase (Sigma-Aldrich, USA) and 9 μg propidium iodide (Invitrogen, USA) for 30 minutes at 4 °C in the dark. DNA content was analyzed using a FACSCalibur flow cytometer (Becton Dickinson, USA).

### Tail vein assay of experimental metastasis

For the tail vein assay [51], 2 × 10^6^ cells were suspended in 0.15 ml of Hank’s solution, filtered to obtain a single-cell suspension, and injected using a 28-gauge needle into a tail vein of six-week-old CB.17 *scid* mice. Mice were sacrificed after 6–8 weeks or when they exhibited signs of disease (weight loss and decreased grooming). Animals were necropsied and lungs and any other organs that showed gross tumors were fixed, paraffin embedded, and stained with hematoxylin and eosin for examination by a pathologist. The number of tumors in lung, heart, kidney, rib, back (near the injection site), leg, and foot was scored. Representative lung tissue sections were imaged using an Aperio Scanscope eSlide capture device and analyzed using Aperio Precision image analysis software.

### cDNA microarray analysis

Gene expression profiling was performed using an Affymetrix Gene Chip Human Genome U133 Plus 2.0 Array, which contains 54,000 probe sets representing 38,500 well-characterized human genes (Affymetrix, Santa Clara, CA). Total RNA was isolated from triplicate cultures using the TRIzol method. After passing a quality control measurement, RNA was amplified and labeled using the Affymetrix 3’ IVT Express kit and hybridized to the array for 16 hours. This was followed by washing and staining using the Affymetrix fluidics station 450. Arrays were scanned using an Affymetrix 3000 7G plus instrument. AGCC software was used to generate .cel files. CHP files were generated with the mas5 method using the Affymetrix Expression Console. Data were imported into the Agilent Gene Spring GX software for further analysis. Statistical significance of inter-group differences was evaluated by a t-test with Bonferroni correction. Unsupervised hierarchical clustering analysis was performed using Spotfire 5.0 software (TIBCO, Somerville, MA). The row dendrogram was generated using Ward’s clustering method with a half square Euclidean distance measure. The column dendrogram was generated using the single linkage clustering method and a Euclidean distance measure. Gene ontology analysis was based on David 6.7 [52, 53] (http://david.abcc.ncifcrf.gov).

### Ethics statement

Ethics approvals were obtained from the Institutional Animal Care and Use Committee of Georgia Regents University (Protocol Number 2008–0155) or by the Experimental Animal Care and Use Committee of Sun Yat-Sen University (Protocol Number 201306). All experiments were conducted in accordance with the institutional guidelines of Sun Yat-Sen University and Georgia Regents University for the care and use of experimental animals.

## Additional files

Additional file 1: Table S1. - Comparison of the genes altered by transgelin overexpression in RKO and DLD-1 cells. Table shows gene symbols, gene names, expression fold changes obtained by cDNA microarray and qPCR in RKO and DLD-1 cells. (DOCX 16 kb) Additional file 2: Table S2. - Primers for qPCR. Table shows gene symbols and primer sequences. All primers are written in 5’ to 3’ direction. (DOC 78 kb)
