# Supplementary material for: Transgelin increases metastatic potential of colorectal cancer cells in vivo and alters expression of genes involved in cell motility
Source: BMC Cancer. 2016 Feb 4;16:55. doi: 10.1186/s12885-016-2105-8 (PMC4741053; doi:10.1186/s12885-016-2105-8)
Supplement: Additional file 1: Table S1. — - Comparison of the genes altered by transgelin overexpression in RKO and DLD-1 cells. Table shows gene symbols, gene names, expression fold changes obtained by cDNA microarray and qPCR in RKO and DLD-1 cells. (DOCX 16 kb) [file 12885_2016_2105_MOESM1_ESM.docx]

**Table S1 – Comparison of the genes altered by transgelin overexpression in RKO and DLD-1 cells**

| Rank | Symbol | Gene name | Affymetrix  fold up RKO | *P* value | qPCR fold up RKO | *P* value | qPCR  fold up DLD-1 | *P* value | Function |
| --- | --- | --- | --- | --- | --- | --- | --- | --- | --- |
| 1 | HOOK1 | Hook homolog 1 | 95.7 | 0.011 | 222.8 | 0.004 | 23.3 | 0.014 | actin binding |
| 2 | SDCCAG8 | serologically defined colon cancer antigen 8 | 85.8 | 0.023 | 294.1 | 0.006 | 24.5 | 0.008 | microtubule organizing center |
| 3 | ENAH | enabled homolog | 17.7 | 0.045 | 14.9 | 0.022 | 6.8 | 0.023 | actin binding |
| 4 | TNS1 | Tensin 1 | 14.3 | 0.014 | 5.6 | 0.029 | 5.05 | 0.032 | actin binding |

| Rank | Symbol | Gene name | Affymetrix  fold down  RKO | *P* value | qPCR  fold down RKO | *P* value | qPCR  fold down DLD-1 | *P* value | Function |
| --- | --- | --- | --- | --- | --- | --- | --- | --- | --- |
| 1 | EMB | embigin | 15.9 | 0.005 | 1.8 | 0.053 | 9.9 | 0.012 | cell adhesion |
| 3 | BCL11B | B-cell CLL/lymphoma 11B (zinc finger protein) | 12.0 | 0.0004 | 12.1 | 0.031 | 5.0 | 0.021 | nucleic acid binding |
| 5 | PTPRD | protein tyrosine phosphatase, receptor type, D | 11.0 | 0.021 | 55.7 | 0.042 | 3.3 | 0.036 | phosphoprotein phosphatase |
