# Supplementary material for: Transgelin increases metastatic potential of colorectal cancer cells in vivo and alters expression of genes involved in cell motility
Source: BMC Cancer. 2016 Feb 4;16:55. doi: 10.1186/s12885-016-2105-8 (PMC4741053; doi:10.1186/s12885-016-2105-8)
Supplement: Additional file 2: Table S2. — - Primers for qPCR. Table shows gene symbols and primer sequences. All primers are written in 5’ to 3’ direction. (DOC 78 kb) [file 12885_2016_2105_MOESM2_ESM.doc]

**Table S2** - primers for qPCR

| Gene | Forward Primer (5’-3’) | | Reverse Primer (5’-3’) |
| --- | --- | --- | --- |
| *TAGLN* | GTTCCAGACTGTTGACCTCTTT | | CTGCGCTTTCTTCATAAACC |
| *GAPDH* | ACAGCCTCAAGATCATCAGCAAT | ATGGACTGTGGTCATGAGTCCTT | |
| *HOOK1* | CAGACATTCAATACTGCCTCACC | | CCCCAACATCCTCTTTAATTCGG |
| *SDCCAG8* | AAGTCCCCGGAGAACTCTACC | | ACATCGCCTTCTTTCAGGGC |
| *ENAH* | TCTATCACCATACAGGCAACAAC | | GCACAGTTTATCACGACCTGA |
| *TNS1* | GTACGTCACAGAGAGGATCATCG | | GCAGGTAGTTGCCTCCATGTT |
| *EMB* | ATGGGGAATCTTACTGGTGCC | | CCCCTCATCTGAGTGCTTCTTT |
| *PTPRD* | CTCCAAGGTTTACACGAACACC | | AGTCCGTAAGGGTTGTATTCTGA |
| *BCL11B* | GGTGCCTGCTATGACAAGG | | GGCTCGGACACTTTCCTGAG |
